# To Physicians

**Published:** 1871-10

**Authors:** 


### The Bistoury.

ELMIRA, N. Y., OCTOBER, 1871.

The BISTOURY is published Quarterly, upon the 1st of April, July, October and January, at Fifty Cents a year, in advance.

### TO PHYSICIANS.

It will be seen that we are bestowing more than our usual amount of labor upon our "MEDICAL ITEMS AND NEWS." We do this for the reason that more than one-third of our patrons are physicians, and we find that a majority of our new subscribers come from among the profession.

We give a short and comprehensive digest of everything of any value to the physician, taken from twenty of the best medical publications in the world. We select with care, condensing an article of several pages in such a manner as to give all its points in as many lines. In this manner we give the physician the benefit of an immense amount of reading, that would require of him much time and a great outlay of money to procure, all for the very small sum of fifty cents.

We send out quite a large number of extra copies of our journal, addressed to different members of the profession, who are not subscribers, but whom we hope will become so after having had an opportunity of examining it.

It is not only our aim to give the physician the latest medical items and news, but also to elevate the legitimate practice of medicine, by warning the non-professional reader against all forms of quackery and humbuggery.

We inclose a blank and envelope to every non-subscriber, by means of which they can readily have their names placed on our list, if they feel so disposed. Subscribers whose subscriptions have expired, will be informed of the fact and invited to renew, by the same gentle reminder.

---

PERSONAL,—RATHER.—Mr. L. S. Buck, the traveling agent for Herring, Farrel & Sherman's fire and burglar proof safes, has been sojourning in our city for several days. He not only has the best safe in the market, but knows how to sell them. As a gentleman and agent he is a marvelous success.

